# Adipokine regulation of immune cell polarization in oral cancer and its impact on chemotherapy sensitivity

**DOI:** 10.3389/fonc.2026.1954382

**Published:** 2026-09-30

**Authors:** Puguan Wang, Jiakai Qiao, Junnan Qi, Huen Li, Jiatong Li, Kunyu Lin, Bei Chang

**Affiliations:** 1 Jilin Provincial Laboratory of Biomedical Engineering, Jilin University, Changchun, China; 2 Hospital of Stomatology, Department of Pediatric Dentistry, Jilin University, Changchun, China

**Keywords:** adipokines, chemotherapy resistance, immune cell polarization, oral squamous cell carcinoma, tumor microenvironment, tumor-associated macrophages

## Abstract

Oral squamous cell carcinoma (OSCC) is the most common malignant tumor in the head and neck region. In locally advanced patients, neoadjuvant chemotherapy centered on cisplatin and 5-fluorouracil often leads to primary/acquired resistance, with a 5-year survival rate of less than 50%. Tumor microenvironment (TME) immunosuppressive remodeling is the core cause of chemotherapy failure. Adipokines secreted by cancer-associated adipocytes (CAAs) are the key mediators that link metabolic disorders, immune polarization and chemotherapy resistance. The adipokines leptin, adiponectin, chemerin, resistin, and GDF15 signal through pathways such as JAK2/STAT3, PI3K/AKT, NF-κB, and PPARγ to bidirectionally regulate the polarization phenotypes of tumor-associated macrophages (TAMs), myeloid-derived suppressor cells (MDSCs), CD4^+^/CD8^+^ T cells, and NK cells. Pro-cancer adipokines induce M2-type TAMs, MDSCs expansion, Th2/Th17 polarization, and effector T cell exhaustion, constructing an immunosuppressive microenvironment, upregulating DNA damage repair, drug efflux, and anti-apoptotic genes, and reducing cisplatin/5-FU chemosensitivity. The tumor-suppressive adipokine adiponectin promotes M1 macrophage and CD8^+^ effector T cell infiltration, reverses immunosuppression, and sensitizes chemotherapy. This article systematically reviews the origin and classification of adipokines, the polarization patterns of immune cells in the OSCC microenvironment, the molecular pathways by which various adipokines regulate immune polarization, and the complete mechanism by which immune polarization mediates chemotherapy resistance. It summarizes the value of clinical markers and targeted reversal strategies, points out current research bottlenecks, and looks forward to the translational prospects of metabolism-immunotherapy combined with chemotherapy, aiming to provide new metabolism-immune targets for overcoming chemotherapy resistance in oral cancer.

## 1 Introduction

### 1.1 Clinical treatment dilemmas of oral cancer and the current situation of chemotherapy resistance

Oral squamous cell carcinoma accounts for more than 90% of oral malignancies (1). Smoking, alcohol consumption, betel nut chewing, HPV infection, obesity and metabolic disorders are the main high-risk factors (2–5). Clinical stage I/II OSCC can be cured by surgery, but more than 60% of patients are initially diagnosed as locally advanced stage III/IV with cervical lymph node metastasis (6). The treatment is mainly surgery combined with neoadjuvant/adjuvant platinum-based chemotherapy, and patients with advanced metastasis can only rely on palliative chemotherapy to prolong survival (7). Cisplatin (DDP) or carboplatin combined with 5-fluorouracil (5-FU) is the first-line standard regimen, but the objective response rate is only 35% to 48%. More than half of patients develop chemotherapy resistance in the short term, manifested as insignificant tumor shrinkage, recurrence and metastasis, aggravated toxicity, and shortened survival (3, 8).

Conventional chemotherapy resistance mechanisms mostly focus on inherent changes in tumor cells: high expression of the DNA repair gene ERCC1 enhances cisplatin damage repair, overexpression of the ABCB1/P-gp drug efflux pump reduces intracellular drug concentration, upregulation of the anti-apoptotic protein Bcl-2/MCL1, and maintenance of stemness of tumor stem cells, etc. However, single-cell sequencing of clinical samples has confirmed that immune remodeling in the tumor microenvironment has a much greater impact on chemosensitivity than mutations in tumor cells themselves. After neoadjuvant chemotherapy, the infiltration density of M2-type TAMs, MDSCs, and regulatory T cells (Treg) in drug-resistant OSCC tissues increased significantly, and M1 anti-tumor macrophages and functional CD8^+^ T cells were depleted, creating a dual microenvironment of “immune desert + immunosuppression”. Even if chemotherapy drugs enter the tumor, they fail to induce immunogenic cell death (ICD), ultimately leading to treatment failure.

Obesity, as an independent high-risk factor for OSCC, has a 42% lower chemotherapy response rate and a 2.1-fold higher recurrence risk in obese patients with a BMI of ≥28 compared to those with normal weight (5). In obese patients, there is extensive infiltration of adipose tissue around the oral mucosa and submandibular glands into the tumor stroma, differentiation into CAAs, and continuous release of hundreds of adipokines, free fatty acids (FFA), and inflammatory mediators, forming a unique metabolic immune microenvironment (9, 10). Clinical correlation analysis confirmed that the levels of serum leptin, chemerin and resistin in OSCC patients were positively correlated with chemotherapy resistance, lymph node metastasis and poor prognosis; Adiponectin levels were significantly negatively correlated with chemotherapy response rate and overall survival, suggesting that adipokines are the core mediators of metabolic disorders mediating chemotherapy resistance in oral cancer.

### 1.2 Research progress on adipokines and tumor immune polarization

Adipose tissue is no longer regarded merely as an energy storage organ but as the largest endocrine organ in the body (11–13) divided into white adipose tissue (WAT), brown adipose tissue (BAT), and tumor stromal CAAs are formed by dedifferentiation of mature adipocytes, with significantly enhanced lipolysis activity and secretion of two types of adipokines: ① Pro-tumor adipokines: leptin, chemerin, resistin, IL-6, VEGF; ② Anti-tumor adipokines: adiponectin, GDF15 (bidirectional action), visfatin (bidirectional action) (14). Adipokines act on tumor cells and infiltrating immune cells through autocrine, paracrine, and endocrine pathways, regulating cell proliferation, apoptosis, lipid metabolism, cytokine secretion, and reshaping the polarization phenotype of immune cells.

Immune cell polarization is at the core of immune microenvironment plasticity: Macrophages are classified into classically activated M1 type (anti-tumor, secreting TNF-α, IL-12, IFN-γ, strong antigen-presenting ability, killing tumor) and alternatively activated M2 type (pro-tumor, secreting IL-10, TGF-β, CCL22, inhibiting effector T cells, promoting angiogenesis, invasion resistance); CD4^+^ T cells are classified as Th1 (anti-tumor), Th2, Th17, and Treg (immunosuppressive); CD8^+^ T cells are divided into functional effector T cells and exhausted PD-1^+^TIM-3^+^ T cells; MDSCs and NK cells also have tumor-promoting/tumor-suppressing polarization subtypes.

Existing studies in breast cancer, lung cancer and pancreatic cancer have confirmed that leptin induces TAMs M2 polarization and MDSCs proliferation, while adiponectin promotes M1 polarization (15, 16). However, there is still a gap in the study of systemic mechanisms for OSCC: The special anatomical location of maxillofacial adipose tissue, the continuous stimulation of chronic oral inflammation, and the involvement of microbiota in metabolic regulation make the metabolic-immune axis of OSCC tissue-specific. The lack of a complete signaling axis for adipokines-immune polarization-chemotherapy resistance, and the absence of *in vivo* and *in vitro* validation systems for targeted adipokines combined with chemotherapy, limits clinical translation. This regulatory network is depicted in **Figure 1**.

**Figure 1:**
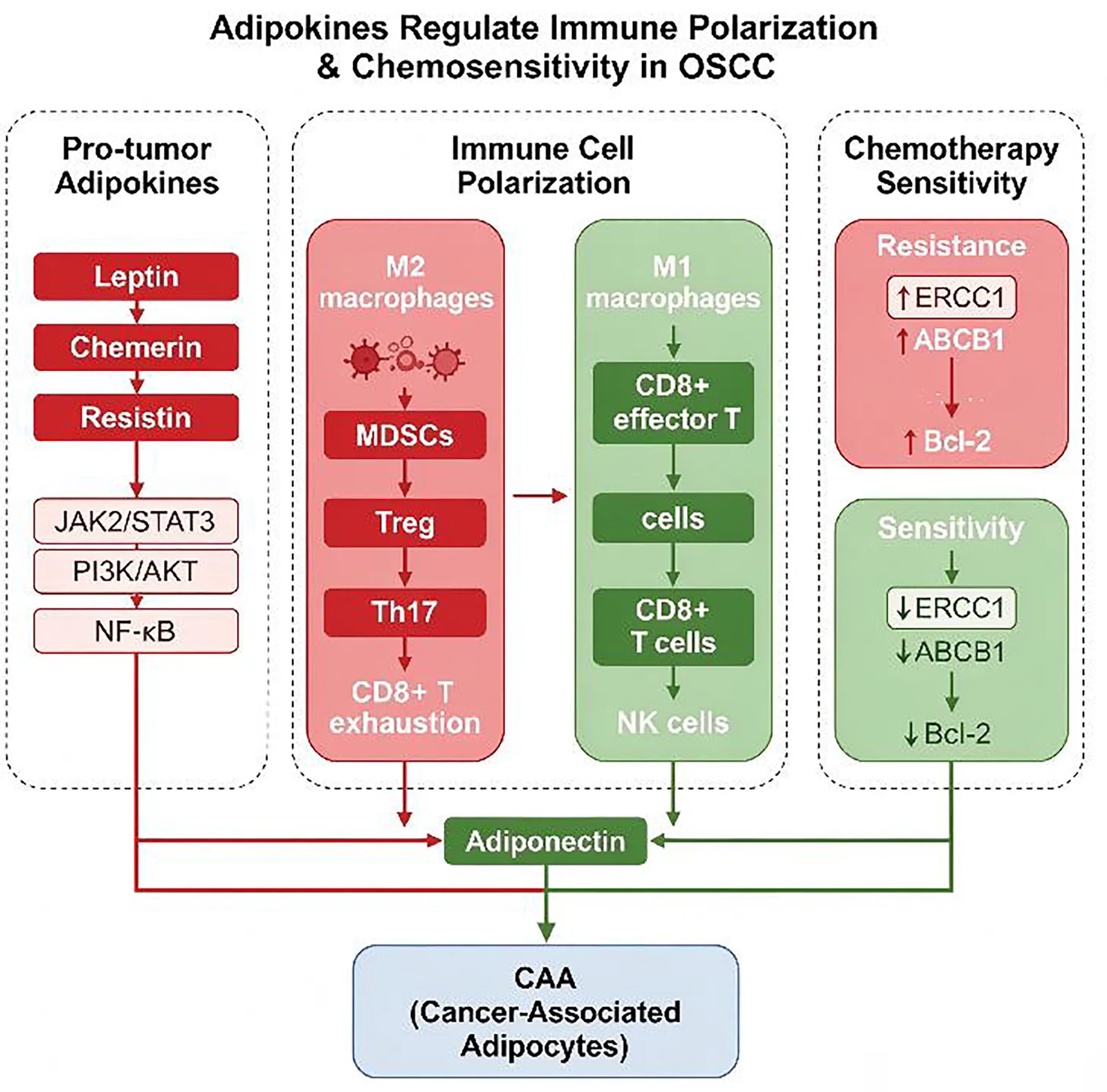
Adipokine regulation of immune polarization and chemosensitivity in OSCC. Upstream, CAAs and adipocytes release pro-tumor adipokines (leptin, chemerin, resistin, IL-6) and the anti-tumor adipokine adiponectin. These converge on the downstream nodes STAT3, NF-κB, PI3K/AKT, and AMPK: pro-tumor adipokines activate JAK2/STAT3, PI3K/AKT, and NF-κB to drive M2 macrophage polarization, MDSC/Treg expansion, and CD8^+^ T-cell exhaustion, upregulating ERCC1, ABCB1, and Bcl-2 to promote chemotherapy resistance, whereas adiponectin activates AMPK to reverse this program and sensitize chemotherapy. CAA, cancer-associated adipocyte.

### 1.3 Study significance and innovation points

By systematically integrating peer-reviewed basic and clinical evidence to construct a complete regulatory network of “CAAs-adipokines-immune cell polarization-chemosensitivity”, the innovations are as follows:

This article is a narrative review rather than a systematic review. To reduce the risk of a biased literature view, we searched PubMed, Web of Science, and Scopus for English-language articles published up to August 2026 using combinations of the terms oral squamous cell carcinoma, adipokine, leptin, adiponectin, chemerin, resistin, tumor microenvironment, immune polarization, chemotherapy resistance, and immune checkpoint inhibitor. The search terms were combined with Boolean operators and adapted to each database. Two authors independently screened titles and abstracts, discrepancies were resolved by consensus, and the reference lists of eligible articles were hand-searched. Any uncertainty about eligibility was resolved by discussion between the two screeners before a study was included or excluded, so that the review reflects a consensus synthesis of the available evidence rather than a pooled quantitative estimate. Mechanisms that are directly proven in OSCC are distinguished in the text from those that have been extrapolated from other adipocyte-rich cancers, and the latter are explicitly labeled as extrapolated.

1. Focusing on the oral cancer-specific microenvironment, compare the bidirectional regulatory differences of different adipokines on the polarization of TAMs, T cells, and MDSCs;
2. Clarify the downstream molecular pathways mediated by immune polarization for cisplatin/5-FU resistance, and distinguish the direct effects of tumor cells from the indirect effects of the immune microenvironment;
3. Summarize the preclinical evidence of adipokines as potential biomarkers for predicting the efficacy of chemotherapy and propose future clinical validation directions;
4. Propose a new strategy for reversing drug resistance by targeting adipokine signaling pathways, reprogramming immune polarization, and combining chemotherapy, providing a theoretical basis for individualized treatment of obesity-related oral cancer.

## 2 Basic theory of adipocytes and adipokines in the tumor microenvironment

### 2.1 Formation and biological characteristics of cancer-associated adipocytes

When OSCC cells invade the maxillofacial subcutaneous and submucosal adipose tissue, tumor-derived IL-6, TNF-α, and TGF-β induce dedifferentiation of mature adipocytes into spindle-shaped cancer-associated adipocytes (CAAs) with reduced lipid droplets, upregulated ATGL/HSL, and massive release of free fatty acids (FFAs) and adipokines (17); CAAs then acquire fibroblast-like motility, infiltrate the tumor stroma, and wrap tumor cells to form a metabolic barrier (18).

CAAs have three core functions: ① supplying FFAs for CD36-mediated uptake and fatty acid oxidation in tumor cells; ② secreting adipokines that reshape the microenvironment; and ③ recruiting monocytes and myeloid cells to induce immunosuppressive polarization (17). Immunofluorescence shows that CAAs localize at the invasive front together with M2-type TAMs and MDSCs, forming drug-impenetrable adipose-immuno-tumor units that act as resistance refuges (18, 19). In obesity, expanded WAT and rising circulating pro-inflammatory adipokines multiply stromal CAAs, amplifying immunosuppression and poor chemotherapy response (3, 5, 20).

Beyond cytokine secretion, cancer-associated adipocytes are metabolically active partners that engage in bidirectional lipid exchange with tumor cells. Tumor-derived signals activate adipose triglyceride lipase (ATGL) and hormone-sensitive lipase (HSL), which hydrolyze stored triglycerides and release free fatty acids (FFAs) into the stroma (21). These FFAs are imported into OSCC cells mainly through the fatty acid translocase CD36 and are bound intracellularly by fatty acid-binding protein 4 (FABP4), which channels them into fatty acid oxidation (FAO) to sustain rapid proliferation, migration, and metastasis (22, 23). Adipocyte-tumor metabolic coupling therefore depends on a coordinated lipolysis-FFA-CD36/FABP4-FAO axis that operates in parallel with adipokine signaling, so the simplistic notion of a CAA-derived adipokine should be replaced by an integrated model in which adipokines, free fatty acids, cytokines, extracellular vesicles, and metabolic substrates act together (23). Adipocyte-derived lipids also compete with infiltrating lymphocytes for metabolic fuels, further tipping the local niche toward immune suppression (23). CAAs also engage in crosstalk with macrophages, T cells, NK cells, cancer-associated fibroblasts, and endothelial cells, further amplifying the immunosuppressive and chemotherapy-resistant niche (17, 24, 25). Clinically, the abundance of these metabolically active CAAs at the tumor invasive front correlates with poor response to neoadjuvant chemotherapy, consistent with a metabolism-driven resistance program (10). Where direct OSCC evidence is unavailable, these mechanisms are extrapolated from ovarian, breast, and hematological malignancies (10, 23).

### 2.2 Classification, structure and receptor expression of adipokines (core factors related to OSCC)

#### 2.2.1 Pro-tumor adipokines

Leptin, a 16-kDa adipokine, signals through Ob-R on OSCC cells, macrophages, T cells, and MDSCs; elevated in obesity and highly expressed in the OSCC stroma, it drives JAK2/STAT3- and PI3K/AKT-mediated immunosuppressive polarization and is associated with lymph node metastasis and chemotherapy resistance (2, 15, 16, 26).

Chemerin, secreted by adipocytes and dendritic cells, acts through ChemR23 on TAMs, NK cells, and OSCC epithelium (19, 27, 28); serum chemerin is markedly higher in drug-resistant OSCC and promotes M2 polarization and cisplatin resistance via STAT3/NLRP3 (27, 29).

Resistin activates NF-κB, drives monocyte differentiation into M2 macrophages, upregulates IL-10 and TGF-β, and inhibits CD8^+^ T-cell infiltration; serum resistin is an independent prognostic risk factor for locally advanced OSCC (30, 31).

CAA-derived IL-6 and VEGF sustain STAT3 activation, a central hub linking lipid metabolism, immune polarization, and chemotherapy resistance (30, 31).

#### 2.2.2 Anti-tumor adipokines

Adiponectin, reduced in obesity, activates AMPK through AdipoR1/R2, inhibits STAT3 and NF-κB, induces M1 macrophage polarization, restores CD8^+^ T-cell function, and sensitizes platinum chemotherapy (15, 32); omentin-1 shows similar tumor-suppressive properties (33).

GDF15 acts bidirectionally: macrophage-derived GDF15 promotes antigen presentation and CD8^+^ infiltration, whereas high circulating free GDF15 induces MDSC expansion and T-cell exhaustion (34); dysregulated adipokines are also observed in other obesity-related malignancies (35).

### 2.3 Ways adipokines are delivered in the OSCC tumor microenvironment

1. Paracrine: CAAs release adipokines locally, directly acting on interstitial infiltrating immune cells, which is the most important pathway for regulating immune polarization (2).
2. Autocrine: Adipokines bind to CAAs’ own receptors, promoting lipolysis and the release of more adipokines, forming a positive feedback loop (36).
3. Endocrine circulation: Leptin and adiponectin secreted by adipose tissue throughout the body reach tumor tissue via the bloodstream, remotely regulating systemic immune homeostasis (37, 38).
4. Exosome transport: CAAs secrete exosomes that encapsulate adipokines, microRNAs, and stably deliver them to distant immune cells, expanding the range of immunosuppression (24).

## 3 Polarization phenotype of immune cells in the microenvironment of oral cancer and regulation of chemosensitivity

TAMs are the highest proportion of immune cells in the stroma of OSCC (40% to 60% of infiltrating immune cells), highly malleable, and microenvironmental signals determine M1/M2 typing, directly regulating the effect of chemotherapy (18, 39).

### 3.1 Tumor-associated macrophage M1/M2 polarization and chemotherapy response

#### 3.1.1 M1-type macrophages: chemotherapy-sensitizing phenotype

LPS, IFN-γ, and adiponectin stimulate monocytes to differentiate into M1 macrophages, with high expression of characteristic markers CD80, CD86, MHC-II and secretion of IL-12, TNF-α, IFN-γ (32, 40). Functions: ① Direct phagocytosis of apoptotic tumor cells; ② Highly efficient antigen presentation, recruitment and activation of CD8^+^ cytotoxic T cells; ③ Induce chemotherapy-mediated immunogenic cell death (ICD), release ATP, HMGB1 damage-associated molecular patterns (DAMPs), and amplify systemic anti-tumor immunity; ④ Down-regulate the ABCB1 drug efflux pump, inhibit tumor DNA repair, and enhance cisplatin cytotoxicity (3, 8). Preclinical evidence suggests that M1 polarization is associated with enhanced chemosensitivity, although systematic clinical validation in OSCC patients remains limited (18, 40).

#### 3.1.2 M2-type macrophages: chemotherapy-resistant core immune cells

IL-4, IL-13, leptin, and chemerin induce monocyte differentiation of M2-type TAMs, markers CD163, CD206, CD209, high secretion of IL-10, TGF-β, CCL22 (18, 39). Mechanisms of multidrug resistance:

1. Immunosuppression: IL-10 inhibits M1 differentiation, blocks CD8^+^ T cell proliferation and granzyme B secretion; TGF-β induces Treg amplification to form a local immunosuppressive barrier, and chemotherapy-killed tumor cells fail to activate anti-tumor immunity (37, 41).
2. Protecting tumor stem cells: M2 secretes cytokines that upregulate SOX2 and OCT4 stemness genes. Stem cells are insensitive to cisplatin DNA damage. Residual stem cells after chemotherapy can lead to recurrence (3).
3. Metabolic protection: M2 synergizes with CAAs to release FFA, enhancing FAO metabolism in tumor cells, high expression of antioxidant Nrf2, eliminating cisplatin-induced reactive oxygen species (ROS), reducing oxidative damage (2, 18).
4. Upregulation of drug-resistant proteins: Continuous activation of STAT3 promotes transcription of ERCC1 and ABCB1, accelerates cisplatin drug excretion and DNA damage repair (8, 19).
5. Accumulating evidence indicates that M2-type TAMs are significantly enriched in drug-resistant OSCC tissues and are associated with tumor residue and lymph node metastasis (39).

### 3.2 The effect of T lymphocyte polarization typing on chemosensitivity

#### 3.2.1 CD4^+^ T cell subsets

1. Th1 cells: Secrete IFN-γ, IL-2, assist CD8^+^ T cells in killing tumors, promote M1 polarization, enhance the ICD effect of chemotherapy; Adipokine adiponectin can induce Th1 differentiation and improve chemotherapy response (32).
2. Th2/Th17 cells: Leptin and chemerin promote Th17 polarization through the PPARγ pathway, secrete IL-17A/C, induce the inflammatory suppressive microenvironment, promote M2 TAMs infiltration, and weaken the efficacy of cisplatin (19, 37). Th17 polarization has been associated with immunosuppression and poorer chemotherapy outcomes in preclinical models (37).
3. Treg cells (Foxp3^+^): M2 secretes TGF-β to induce Treg proliferation; Tregs express CTLA-4 and PD-L1 and inhibit effector T cell function; Obese OSCC patients have elevated circulating leptin, a significantly increased proportion of Treg in peripheral blood, and an increased risk of chemotherapy resistance (42).

#### 3.2.2 CD8^+^ cytotoxic T cells

Functional CD8^+^ T cells (GranB^+^IFN-γ^+^) are key to clearing minimal residual foci after chemotherapy. Adipokine imbalance induces CD8^+^ T cell exhaustion: Leptin-mediated high expression of CD36, excessive uptake of FFA causes mitochondrial dysfunction, upregulation of PD-1 and TIM-3 exhaustion markers, depletion of IFN-γ and granzyme secretion, loss of tumor-killing ability (41). Even if chemotherapy kills most tumor cells, residual lesions cannot be immunologically cleared, leading to rapid recurrence and metastasis (18).

### 3.3 Myeloid-derived suppressor cells, NK cell polarization and chemotherapy resistance

1. MDSCs: Divided into monocytic M-MDSC and granulocytic PMN-MDSC, leptin, resistin, and CAAs secrete CCL9 to recruit bone marrow MDSCs into the tumor stroma via the CCR1 axis (30). MDSCs secrete arginase 1 (Arg-1), reactive oxygen species, inhibit T cell proliferation, promote M2 polarization, and construct a strong immunosuppressive microenvironment (18, 43). Preclinical studies have demonstrated that targeting MDSC infiltration can reverse cisplatin resistance.

2. NK cells: Normal NK cells secrete perforin to kill tumors; chemerin and leptin induce lipid overload in NK cells, cytotoxicity decreases, and IFN-γ secretion decreases (19). Adiponectin restores NK cell function, complements chemotherapy to clear circulating tumor cells and reduces the risk of distant metastasis (32).

The role of NK cells extends well beyond perforin release. NK-cell cytotoxicity is governed by a balance between activating receptors (NKp46, NKG2D, and DNAM-1) and inhibitory checkpoints (TIGIT, PD-1, and TIM-3); sustained exposure to pro-tumor adipokines and lipids shifts this balance toward exhaustion, with reduced interferon-γ (IFN-γ), perforin, and granzyme B secretion (44). Tumor cells frequently downregulate the NKG2D ligands MICA/B and the DNAM-1 ligands CD155/CD112, while adipokines reinforce inhibitory signaling, together impairing NK-cell recognition of malignant cells (44). It is also necessary to separate reduced NK-cell infiltration from functional NK-cell exhaustion and metabolic dysfunction. Obesity-associated lipids and hypoxia drive lipid accumulation in NK cells, impair mitochondrial metabolism, and limit nutrient utilization, which can paralyze NK-cell trafficking and cytotoxicity even when NK cells remain present in the tumor bed (45). Leptin, chemerin, and transforming growth factor-β (TGF-β) suppress NK-cell chemotaxis and effector function, whereas adiponectin restores NK-cell activity and supports chemotherapy-mediated clearance of circulating tumor cells (19, 32). Therapeutic strategies that normalize lipid metabolism, such as PPARα/δ blockade, can restore NK-cell cytotoxicity and synergize with conventional chemotherapy (45). Because NK-cell status reflects both recruitment and functional competence, future biomarkers should capture NK-cell density together with activating and inhibitory receptor profiles and metabolic fitness (44).

## 4 Core adipokines regulate the molecular mechanism of oral cancer immune cell polarization mediating chemotherapy resistance

### 4.1 Leptin/Ob-R axis: dominant tumor-promoting immune polarization and platinum resistance

Leptin is the most crucial adipokine for chemotherapy resistance in obese OSCC (15, 26). Its pathways of action are divided into two: directly regulating the polarization of immune cells and indirectly amplifying drug resistance in tumor cells (2, 16).

#### 4.1.1 Leptin-induced TAMs M2 polarization molecular pathway

Leptin binds to the macrophage Ob-R receptor, activates JAK2 phosphorylation, and follows two parallel pathways downstream (15):

1. JAK2/STAT3 pathway: STAT3 enters the nucleus, transcriptionally upregulates CD163, CD206, IL-10, TGF-β M2 characteristic genes; At the same time, NF-κB p65 activity is inhibited, IL-12 and TNF-α M1 gene transcription is blocked, and monocytes are completely tilted towards the M2 phenotype (29).
2. PI3K/AKT/mTOR pathway: AKT phosphorylation activates mTORC1, promoting lipid synthesis and lipid droplet accumulation, macrophage metabolism reprograms to fatty acid-dependent, further amplifying immunosuppressive cytokine secretion.
3. *In vitro* co-culture experiments have demonstrated that leptin significantly promotes M2 polarization and enhances the resistance of OSCC cells to cisplatin, while STAT3 pathway inhibition reverses this effect (16, 26).

#### 4.1.2 Leptin regulates T cell and MDSCs polarization

1. CD4^+^ T cells: Leptin activates the STAT3/PPARγ pathway, promotes Th17 differentiation, IL-17C massive secretion, upregulates PD-L1 in OSCC cells, and inhibits CD8^+^ infiltration (37).
2. CD8^+^ T cells: Ob-R-mediated upregulation of CD36, massive uptake of CAAs to release FFA, mitochondrial ROS accumulation, increased PD-1/TIM-3 expression, T cell exhaustion (18).
3. MDSCs: Leptin stimulates proliferation of myeloid cells in the bone marrow, upregulates Arg-1 and iNOS, inhibits T cell function, and creates multiple immunosuppressive superposition effects (26).

#### 4.1.3 Leptin directly enhances intrinsic chemotherapy resistance in OSCC cells

Leptin acts on tumor cell Ob-R, STAT3 nuclear transcription: ① ERCC1 (cisplatin DNA repair), ABCB1 (drug efflux pump); ② Anti-apoptotic MCL1, Bcl-2; ③ Stemness SOX2, OCT4. Multiple pathways work together to reduce cisplatin and 5-FU cytotoxicity, creating a dual resistance of “immunosuppression + inherent tumor resistance” (3).

### 4.2 Chemerin/ChemR23 axis: pro-inflammatory immune polarization accelerates chemotherapy failure

Chemerin is significantly highly expressed in the stroma of OSCC tumors and in the serum of obese patients, and regulates immune polarization through the STAT3/NLRP3 dual pathway: (27–29).

1. Macrophage polarization: Chemerin binds to ChemR23 to activate STAT3, inducing M2-type TAMs and secreting IL-6, TNF-α (29). At the same time, NLRP3 inflammasome is activated to release IL-1β, creating a chronic inflammatory microenvironment (46). IL-6 further positively feeds back to activate STAT3, forming a chemerin-STAT3-IL-6 vicious cycle (29).
2. NK cell inhibition: Chemerin inhibits NK cell chemotaxis and perforin secretion, reducing the ability to clear circulating tumor cells.
3. Tumor drug resistance amplification: The IL-6/STAT3 pathway upregulates the ABCG2 drug efflux pump, enhancing 5-FU resistance in OSCC cells (8, 19). Preliminary clinical observations suggest that high chemerin expression may correlate with reduced chemotherapy response, though larger cohort studies are needed (27, 47).

*In vivo* studies using 4NQO-induced OSCC mouse models have shown that targeting chemerin signaling in combination with cisplatin significantly enhances anti-tumor efficacy (28).

### 4.3 Resistin/NF-κB pathway: broad-spectrum immunosuppression and chemotherapy resistance

Resistin mainly activates the NF-κB p65 pathway in macrophages, promotes M2 differentiation in monocytes, and increases the secretion of IL-10 and CCL2 (30, 31). CCL2 recruits more peripheral monocytes to infiltrate the tumor, continuously expanding the M2 pool. NF-κB simultaneously upregulates PD-L1 and DNA damage repair proteins in OSCC cells, synergistically weakening the effect of platinum-based chemotherapy (18, 30). Serum resistin levels have been positively correlated with M2 macrophage infiltration in tumor tissues, suggesting its potential as a predictive biomarker (48).

### 4.4 Adiponectin/AMPK axis: reversing immunosuppression, sensitizing chemotherapy (tumor-suppressive adipokine)

Adiponectin is functionally antagonistic to pro-tumor adipokines, and low expression is a key risk factor for chemotherapy resistance in obese OSCC:

1. Reprogramming TAMs to M1 polarization: Adiponectin binds to macrophage AdipoR1, activates AMPK, inhibits STAT3 and NF-κB activity, downregulates CD163 and IL-10, upregulates CD86, IL-12, and TNF-α, restoring macrophage anti-tumor function (15).
2. Restoring functional CD8^+^ T cells: The AMPK pathway inhibits CD36-mediated lipid overuptake, relieves mitochondrial stress, downregulates PD-1 exhaustion markers, and increases IFN-γ and granzyme B secretion.
3. Direct sensitization of tumor cell chemotherapy: AMPK inhibits the mTOR pathway, downregulates ERCC1 and ABCB1, and promotes cisplatin-induced ROS generation and tumor cell apoptosis. *In vitro* and *in vivo* studies have confirmed that adiponectin supplementation enhances the cytotoxic effects of cisplatin in OSCC models (32, 49).

### 4.5 GDF15 bidirectionally regulates immune polarization — dose- and cell-derived dependence

Endogenous GDF15 in macrophages promotes M1 polarization through NF-κB, enhances phagocytic function, and improves chemotherapy response. But when CAAs and tumor cells secrete high concentrations of circulating free GDF15, they induce MDSCs expansion and T cell exhaustion, weakening the effect of chemotherapy. This bidirectional effect explains the contradictory results of GDF15 expression and prognosis in clinical practice and provides precise ideas for targeted therapy: activating only macrophage GDF15 signaling and inhibiting tumor/adipocyte-derived GDF15 release can synergistically exert anti-tumor effects with chemotherapy (34).

### 4.6 Adipokine cross-regulatory network

Adipokines form a hierarchical cross-regulatory network. Upstream, obesity-driven leptin and resistin promote CAA release of chemerin and IL-6 (30). These pro-tumor inputs converge on two central nodes: STAT3, activated by leptin (JAK2/STAT3), chemerin (ChemR23/STAT3/NLRP3), and IL-6 (autocrine STAT3) (26, 29), and NF-κB, activated by resistin and the NLRP3 inflammasome (30, 46). Downstream, STAT3 and NF-κB cooperatively drive M2 polarization, PD-L1 expression, and DNA-repair/drug-efflux programs, while IL-6 feeds back to further activate STAT3 (18, 29). The negative regulator adiponectin acts through AMPK to block both STAT3 and NF-κB, serving as the core counter-regulatory node of metabolic-immune balance (32, 50). This circuit ultimately tips the microenvironment toward M2/Treg/MDSC dominance and irreversible chemoresistance (18, 51).

### 4.7 Oral microbiome and chronic oral inflammation as an additional metabolic-immune axis

The oral cavity is chronically colonized by a polymicrobial community that is not merely a bystander in OSCC. Oral dysbiosis and periodontal inflammation, driven by pathobionts such as Fusobacterium nucleatum and Porphyromonas gingivalis, activate Toll-like receptor signaling and inflammasome-cytokine pathways that sustain local and systemic inflammation (43). These bacterial products can aggravate adipose tissue inflammation, alter circulating adipokine levels, and reinforce the immunosuppressive polarization of tumor-associated macrophages (52). Periodontal disease and systemic metabolic status may therefore act as important confounders in clinical studies that correlate adipokine concentrations with chemotherapy response (52). In particular, Porphyromonas gingivalis and Fusobacterium nucleatum engage Toll-like receptors 2 and 4 to drive NF-κB and IL-6 signaling, linking local microbial inflammation to systemic adipokine changes (43, 52). Integrating the oral microbiome as an additional axis that connects metabolic dysfunction, adipokine secretion, immune remodeling, and therapeutic response provides a more complete model in which microbial, metabolic, and immune signals converge to shape chemosensitivity in OSCC (43).

### 4.8 Clinical treatment contexts and immune checkpoint inhibitors

Adipokine-mediated resistance should also be interpreted across the full clinical treatment continuum. In locally advanced OSCC, induction chemotherapy, concurrent chemoradiotherapy, and adjuvant therapy expose tumors to cisplatin/5-fluorouracil at different intensities and time points, and adipokine-driven survival signals may differentially blunt each modality (7). In recurrent or metastatic disease, immune checkpoint inhibitors (ICIs) targeting the PD-1/PD-L1 axis are now central to treatment: nivolumab improves survival in platinum-refractory head and neck squamous cell carcinoma, and pembrolizumab, alone or combined with chemotherapy, is a first-line option for recurrent or metastatic disease (53, 54). Adipokines intersect with this pathway at multiple levels, because leptin and chemerin can upregulate PD-L1 on tumor and myeloid cells, promote T-cell exhaustion, and sustain immunosuppressive macrophages, thereby potentially limiting ICI efficacy (18, 19, 41). Because PD-L1 expression is frequently enriched on M2-polarized macrophages in the OSCC microenvironment, adipokine-driven macrophage polarization may partly explain the variable benefit of checkpoint blockade in obese patients with oral cancer (18). Conversely, reducing pro-tumor adipokines or restoring adiponectin signaling may relieve PD-1/PD-L1-mediated immunosuppression, and co-administration of adiponectin receptor agonists or AMPK activators with anti-PD-1 antibodies may represent a rational strategy to sensitize checkpoint blockade (50), while long-term ICI toxicity must be balanced against these metabolic interventions (55).

## 5 Therapeutic strategies targeting adipokines to reprogram immune polarization and reverse chemotherapy resistance in oral cancer

Based on the adipokine regulatory axis, there are four major categories of strategies for reversing resistance in combination with chemotherapy (2):

### 5.1 Inhibiting tumor-promoting adipokine synthesis/neutralizing secretory proteins

1. Leptin/Ob-R pathway blockade: Leptin neutralizing antibodies and Ob-R receptor antagonists inhibit JAK2/STAT3 activation, block M2 polarization. Preclinical animal studies have confirmed that combining leptin pathway blockade with cisplatin significantly reduces tumor burden in obese OSCC models. JAK2 inhibitor (ruxolitinib) is clinically oral, blocks STAT3 downstream of leptin, and simultaneously inhibits MDSCs expansion, suitable for patients with systemic metabolic disorders (26). Leptin/Ob-R antibodies and Ob-R antagonists remain preclinical; the JAK1/2 inhibitor ruxolitinib is clinically approved for myelofibrosis but not for OSCC, and its myelosuppression and gastrointestinal toxicity limit long-term combination with chemotherapy.
2. Chemerin neutralizing antibodies: Block ChemR23 signaling, inhibit NLRP3 inflammasome, reduce IL-6 secretion, reverse macrophage M2 phenotype (27). Preclinical evidence suggests that combining chemerin blockade with platinum-based chemotherapy may reduce residual tumor cells (19, 28). Chemerin/ChemR23 antibodies are still in early preclinical development, with no approved agent and an unestablished safety profile (19, 27).
3. NF-κB inhibitors: Inhibit the downstream pathway of resistin, reduce M2 macrophage recruitment, lower PD-L1 expression, and sensitize chemotherapy (30). NF-κB inhibitors such as bortezomib are clinically approved for hematologic malignancies but are limited by peripheral neuropathy and cytopenias, and their role in OSCC remains unproven (30).

### 5.2 Supplement exogenous tumor-suppressive adipokine adiponectin

Recombinant adiponectin proteins and adiponectin receptor agonists activate the AMPK pathway, with multiple benefits: promoting M1 polarization, restoring CD8^+^ T cell function, directly inhibiting tumor resistance genes (32, 49). However, adiponectin has a short half-life and requires nano-lipid carriers for targeted delivery to the tumor stroma to increase local drug concentration and reduce systemic side effects, which remains a technical challenge for clinical translation. Recombinant adiponectin and AdipoR agonists remain preclinical; their short half-life and off-target AMPK activation require nanocarrier delivery to reduce systemic side effects (32, 50).

### 5.3 Targeted immune polarization reprogramming drugs (combined with metabolic targeting + chemotherapy)

1. M2 to M1 reprogramming drugs: PPARγ inhibitor GW9662 blocks leptin-mediated Th17/M2 polarization. Metformin activates AMPK, mimics adiponectin function, is a clinically common hypoglycemic drug with high safety, and has shown potential in improving chemotherapy response in obese OSCC patients in preliminary studies (2). GW9662 is a preclinical PPARγ antagonist, whereas metformin is clinically available and safe but still lacks randomized evidence for OSCC (2).

2. MDSCs scavengers: COX2 inhibitors reduce adipokine secretion in CAAs, lower MDSC infiltration, and work in synergy with cisplatin to kill tumors (18). COX-2 inhibitors such as celecoxib are clinically approved but carry cardiovascular and gastrointestinal risks (18).

### 5.4 Design ideas for clinical regimens of metabolic immunology combined with chemotherapy

1. High-risk obese OSCC (high leptin, low adiponectin): Neoadjuvant chemotherapy (cisplatin + 5-FU) + metformin + JAK2 inhibitor, preoperative remodeling of the immune microenvironment to increase surgical resection rate.
2. Chemotherapy-resistant recurrent OSCC: Leptin neutralizing antibody + PD-1 immune checkpoint inhibitor + second-line chemotherapy, dual relief of immunosuppression.
3. Low BMI normal metabolism patients: Standard chemotherapy alone, no additional metabolization-targeted drugs required.
4. Adjunctive maintenance therapy: Oral preparations of adiponectin agonists to reduce the risk of postoperative recurrence (currently under preclinical investigation) (2, 26, 32).

## 6 Bottlenecks and challenges in current studies

### 6.1 Limitations of basic mechanism research

1. Insufficient tissue-specific studies: Existing adipokine immune regulation is mostly based on breast cancer and lung cancer, and the synergistic mechanism of OSCC maxillofacial fat, oral microbiota, and chronic inflammation lacks systematic *in vivo* validation. Oral microbiota can regulate CAAs lipolysis and adipokine secretion, but the microbiota-adipokine-immune axis has not been elucidated (18, 43). Single *in vitro* model: Most experiments only use co-culture of tumor cells and macrophages, lacking three-dimensional organoid models including CAAs, T cells, and MDSCs, and unable to restore the complete metabolic immune microenvironment. The mechanism of pathway crossover is ambiguous: leptin, chemerin, and IL-6 share the STAT3 core pathway, the specific downstream branch regulation of each factor is indistinct, and there is a lack of stratified validation in gene knockout animal models (29).
2. Scarce adipokine-targeted drugs: Leptin and chemerin neutralizing antibodies are still in the basic experimental stage, and no clinical preparations have been approved. Systemic inhibition of JAK2 and NF-κB inhibitors has toxic side effects in the blood and gastrointestinal tract and is difficult to combine with chemotherapy for a long time (25, 55)
3. Lack of biomarker standardization: Serum adipokine detection kits are not uniform, and there are significant differences in critical values among different laboratories, which cannot be widely used for clinical stratification. Lack of large-sample prospective cohorts: The existing evidence is largely preclinical or retrospective, and no randomized controlled trials have confirmed that targeted adipokines combined with chemotherapy can improve overall survival in OSCC patients.

### 6.2 Summary of the study conclusions

The study systematically explored the effect of adipokines regulating the polarization of immune cells in oral cancer on chemosensitivity and revealed the key role of adipokines in the occurrence, development and chemoresistance of oral cancer (1). Preclinical evidence indicates that adipokines are significantly highly expressed in oral cancer tissues, and their expression levels are positively correlated with the malignancy of the tumor (1). Further studies have shown that adipokines can regulate the polarization state of tumor-associated macrophages (TAMs) by activating signaling pathways such as PI3K/Akt, promoting their transformation to a pro-tumor phenotype (M2 type), thereby suppressing anti-tumor immune responses and enhancing tumor cell resistance to chemotherapy drugs (16, 26). In addition, adipokines can also affect the immune microenvironment by regulating abnormal lipid metabolism, leading to dysfunction of tumor-infiltrating immune cells and further exacerbating tumor immune escape (2). These findings not only provide a new perspective for understanding the molecular mechanisms of chemotherapy resistance in oral cancer, but also lay a theoretical foundation for developing treatment strategies based on the regulation of adipokines (32).

The significance of the study lies in the first organic combination of adipokines, immune cell polarization and oral cancer chemosensitivity, revealing the complex regulatory network among the three (50). This discovery offers a potential new target for overcoming chemotherapy resistance in oral cancer. For example, by targeting adipokines or their downstream signaling pathways, it is possible to effectively reverse the immunosuppressive tumor microenvironment and enhance the cytotoxic efficiency of chemotherapy drugs against tumor cells. At the same time, the study highlights the importance of multidisciplinary intersections, combining research methods from tumor biology, immunology and metabolomics to provide new ideas for further exploration of the complex pathological mechanisms of oral cancer (2, 18). In conclusion, the study not only expanded the scope of research on adipokines in the field of tumor immunology, but also provided an important theoretical basis for individualized treatment of oral cancer (26).

## 7 Discussion

### 7.1 Full text summary

Adipokines secreted by CAAs are the key bridge connecting metabolic disorders in obesity, OSCC immune polarization and chemotherapy resistance: Pro-tumor adipokines such as leptin, chemerin, and resistin activate STAT3, NF-κB, and PPARγ pathways, induce TAMs M2 polarization, MDSCs and Treg amplification, CD8^+^ T cell exhaustion, construct an immunosuppressive microenvironment, and upregulate DNA repair, drug efflux, and anti-apoptotic genes, dual mediating cisplatin/5-FU chemotherapy resistance (19, 26, 30, 31). Adiponectin acts as a protective adipokine, reversing immunosuppression through the AMPK pathway, reprogramming M1 macrophages with functional cytotoxic T cells, and significantly enhancing chemosensitivity (15, 32).

The combined strategy of targeting the tumor-promoting adipokine pathway, supplementing adiponectin, and reprogramming immune polarization has shown efficacy in reversing chemotherapy resistance *in vitro* and in animal models, providing a promising therapeutic approach for locally advanced, recurrent and metastatic oral cancer (2). Future clinical validation is warranted to translate these preclinical findings into patient benefit.

### 7.2 Future research directions

1. At the basic mechanism level: Construct a three-dimensional OSCC organoid containing CAAs, oral microbiota, and multiple subsets of immune cells, and analyze the transcriptome dynamic changes of adipokines regulating immune polarization in combination with single-cell RNA sequencing. Use leptin and AdipoR1 gene knockout 4NQO oral cancer mice to stratify and verify the specific resistance pathways of each adipokine. Elucidate the interregulatory mechanism between oral periodontal pathogenic bacteria and CAAs adipokines.
2. At the clinical translational level: Conduct multicenter prospective cohorts, unify serum adipokine detection standards, and establish OSCC chemotherapy risk prediction models. Develop a tumor-targeted nanodelivery system to wrap adiponectin and leptin siRNA and reduce systemic toxic side effects. Conduct Phase II clinical studies to verify the real benefits of metformin/JAK2 inhibitors combined with standard chemotherapy in improving survival in obese OSCC patients.
3. At the individualized treatment level: Construct stratified treatment regimens based on BMI, HPV status, serum adipokine profile, and tumor tissue immune polarization markers, and give priority to metabolism-immune-chemotherapy triple regimens for high leptin resistance population to achieve precise individualized treatment of oral cancer.
